# “Bowel prep hyponatremia“ – a state of acute water intoxication facilitated by low dietary solute intake: case report and literature review

**DOI:** 10.1186/s12882-017-0464-2

**Published:** 2017-02-07

**Authors:** Martin Windpessl, Christoph Schwarz, Manfred Wallner

**Affiliations:** 1 Fourth Department of Medicine, Section of Nephrology, Klinikum Wels-Grieskirchen, Grieskirchnerstraße 42, 4600 Wels, Austria; 2 First Department of Medicine, Landeskrankenhaus Steyr, Steyr, Austria

**Keywords:** Bowel preparation, Colonoscopy, Hyponatremia, Water intoxication, Low-solute hyponatremia, Case report

## Abstract

**Background:**

Symptomatic hyponatremia is considered a rare complication of oral bowel preparation for colonoscopy. The pathophysiology underlying this phenomenon has been widely regarded as a mere sequela of excessive arginine vasopressin (AVP) release.

**Case presentation:**

This case describes a 61-year old woman who developed acute hyponatremic encephalopathy when preparing for elective outpatient lower endoscopy. She had had negligible oral solute intake for two days and ingested four liters of clear fluid within two hours. On admission, the patient was agitated and had slurred speech. Treatment with hypertonic saline lead to full recovery. A brisk aquaresis confirmed acute dilutional hyponatremia.

**Conclusion:**

Apart from elevated AVP-levels, the amount and speed of fluid intake and concomitant low-solute intake constitute important risk factors in the development of clinically relevant hyponatremias in patients undergoing colonoscopies. Understanding that the cause of sodium imbalance in this scenario is multifactorial and complex is pivotal to recognizing and ideally preventing this complication, for which we propose the term “bowel prep hyponatremia”.

## Background

Colonoscopy is the gold standard method for colorectal cancer screening with millions being performed each year [1, 2]. A prerequisite for high quality examinations, oral bowel-cleansing preparations have been demonstrated to be safe for use in individuals without significant comorbidities. On rare occasions, however, profound electrolyte abnormalities can ensue [2]. While the role of the various purgatives and the effect of non-osmotic arginine vasopressin (AVP) release as a trigger for hyponatremia in this clinical setting have been extensively discussed, the importance of two other contributory factors, namely preceding low dietary solute intake and the amount and speed of concomitant water intake, has not been appreciated to date. Free water clearance is significantly impaired if solute intake is markedly decreased and electrolyte-free water intake is high, predisposing individuals to dilutional hyponatremia, as exemplified by our case. In all, this scenario is reminiscent of acute hyponatremia in marathon runners. When preparing for colonoscopy, the inevitably restricted diet plays a crucial role in the development of what we propose to call “bowel prep hyponatremia“.

## Case presentation

A 61-year-old woman was admitted because of sudden onset of confusion and slurred speech. In preparation for an elective outpatient colonoscopy she had commenced bowel preparation four hours earlier with sodium picosulfate/magnesium citrate (PICOLAX ®). As instructed, she had ingested two liters of water and two liters of tea, albeit within two hours. Shortly thereafter, she felt nauseous, dizzy and vomited repeatedly. Her husband found her confused with unintelligible speech and unsteady gait and called the ambulance.

On physical examination, the patient appeared agitated and her speech was incomprehensible. She weighed 56 kg and her height was 168 cm (BMI 19.9 kg/m^2^). Vital signs were as follows: Afebrile, blood pressure 132/66 mmHg, pulse 82 beats/min. The patient was clinically euvolemic and could follow verbal commands. No lateralizing signs were found on neurological examination but generalized tremor was present. Acute CT scanning of the brain did not reveal any abnormalities.

Her medical records showed a history of hypothyroidism for which she took levothyroxine. She was a non-smoker and did not drink alcohol. Family history was unremarkable and there was no history of diuretic use or anorexia, as corroborated by her husband.

In the emergency department, biochemistry results were as follows: Serum sodium 122 mmol/l, potassium 3.1 mmol/l, chloride 87 mmol/l, BUN 14.8 mg/dl, creatinine 1.1 mg/dl, uric acid 4.1 mg/dl, glucose 108 mg/dl. Serum osmolality was 251 mOsm/kg. In view of the patient’s symptoms and the clear time of onset, acute hyponatremia was deemed likely and treatment with hypertonic saline (3%) at a rate of 50 ml/h was commenced. Urine osmolality was not done on admission but was 232 mOsm/kg with a urinary sodium of 39 mmol/l when tested two hours later. Thyroid-stimulating hormone was suppressed under replacement therapy. The sodium level increased to 128 mmol/l within the ensuing four hours. In parallel, the patient’s symptoms abated. Twelve hours after admission, she had voided 2600 ml of urine and her mentation and electrolytes had normalized.

On further questioning, it transpired that the patient had had very limited food intake prior to the scheduled procedure. On the day before admission (two days before the endoscopy appointment), her diet consisted of carrot-ginger soup with white bread for lunch and rusk with tea for dinner. The next day, a breakfast consisting of two slices of plain toast and a cup of coffee, followed by a broth at lunchtime, was all she had to eat. No additional salt had been added to her meals. Furthermore, the patient had been taking a nonsteroidal anti-inflammatory drug (NSAID) for the last five days because of shoulder pain (Diclofenac 50 mg bid). A diagnosis of hyponatremic encephalopathy due to acute water intoxication facilitated by poor dietary solute intake was made. On follow-up appointment 1 week later, the patient was well and electrolytes were normal. Adrenal function was tested and found to be intact.

## Discussion and conclusions

Essentially a disorder of water balance, hyponatremia is the most prevalent electrolyte abnormality in clinical practice [3]. Acute hyponatremia is frequently associated with major neurologic manifestations including seizures, coma, permanent brain damage, and death [3, 4]. It is often encountered in hospitalized patients, typically in the postoperative state in the context of pain and nausea, both well-known triggers of non-osmotic AVP-release, and concomitant treatment with electrolyte free water solutions such as 5% dextrose. The development of hyponatremia in the extra-hospital setting is less well recognized. Exercise-induced hyponatremia, for instance following marathon runs, constitutes a prime example; in this setting, high fluid intake and non-osmotic AVP-release are main contributors to acute water intoxication, too [5]. In a chart review involving more than thousand hyponatremic patients presenting to an emergency department, only eleven patients (0.8%) were identified with acute hyponatremia [4].

Colonoscopy remains the method of choice for evaluation of the large intestine [6]. Adequate colonic cleansing is crucial for the quality of the examination and various purgative regimens are available to this end. While polyethylene glycole (PEG) is still considered the prototypical agent, picosulfate/magnesium citrate (PICOLAX ®) was found to be equally effective and is regularly used in Europe [7]. Although generally safe in the healthy population, significant adverse effects have been reported following bowel preparation and encompass pulmonary aspiration, anaphylaxis and electrolyte disturbances, amongst others [8, 9].

Acute water intoxication in preparation for colonoscopy was first reported by Schröppel et al. [9]. Their patient was a 59-year old woman who had ingested 7 liters of fluid over a 7-h period. Her sodium was 122 mmol/l on admission and her clinical course was strikingly similar to our patient’s.

In 2003, Ayus and colleagues reported the clinical course of three patients who developed profound dysnatremias as a complication of elective colonoscopy [10]. While two male patients exhibited significant comorbidities and proceeded to undergo endoscopy, ultimately with a fatal outcome, the index patient was a comparatively healthy woman of 62 years who developed status epilepticus after bowel preparation and was found to have severe hyponatremia (116 mmol/l).

Since then, more than a dozen additional cases of symptomatic “bowel prep hyponatremia” have been reported in the medical literature [11–20]. In these cases, the median patient age was 69.5 years with only two patients younger than 50 years; 17 of 18 patients were female. The role of the various purgatives is discussed extensively in these reports and the principal pathophysiological focus is placed on the risk of volume depletion following bowel preparation triggering AVP-stimulation [19, 21]. Accordingly, patients are generally encouraged to drink fluids liberally [16, 21].

However, information is scarce on two other crucial aspects of renal water handling, namely the amount of dietary solute intake before endoscopy and the characteristics of concomitant fluid ingestion. Specifically, none of the previous reports specified the particular dietary components prior to endoscopy in detail and only limited information is given with regard to the amount and speed of fluid intake (Table 1).

**Table 1:** Characteristics and presentation of patients with symptomatic hyponatremia related to bowel preparation reported in the literature

Reference	Age	Gender	Na Nadir	Presentation	Prep	Time	Diet	U_osm_	U_Na_	U_K_
4	54	f	119	Bizarre behavior	4.5 l PEG	-	-	-	-	-
9	59	f	120	Seizure	3 l PEG-ELS + 4 l tea	7	-	185	22	5
10	62	f	116	Seizure	4 l PEG-ELS + water	-	-	-	-	-
11	75	f	116	Seizure	SP	-	-	-	-	-
11	64	f	111	Seizure	SPS/MC	-	-	-	-	-
11	24	f	132	Seizure	SP	-	-	-	-	-
12	79	f	108	Coma	“cathartics”	-	-	-	-	-
13	73	f	117	Seizure	PEG + 64 ounces of Gatorade	-	-	390	146	35.7
14	80	f	110	Seizure	SPS/MC	-	-	-	-	-
15	70	f	110	Seizure	4 l PEG + 3 l water	-	-	-	-	-
15	65	f	127	Seizure	4 l PEG	-	-	-	-	-
16	34	m	117	Encephalopathy	Bis + Man + > 6 l water	-	small breakfast, broth	-	-	-
17	57	f	120	Rhabdomyolysis	SPS/MC + Bis	-	liquid diet for 3 days	-	67	24
18	69	f	113	Seizure	4 l PEG	-	-	344	122	28.5
19	76	f	112	Seizure	SPS/MC	-	-	370	-	-
Present case	61	f	122	Encephalopathy	2 l SPS/MC + 2 l tea	2	broth, rusk for 2 days	232	39	-

*Abbreviations*: *Na-Nadir* lowest serum sodium concentration recorded (mmol/l), *Prep* type of bowel preparation used and amount of fluid ingested in preparation for endoscopy (l), *time* speed of fluid ingestion (hours), *U osm* urine osmolality (mosm/l), *U*_*Na*_ urine sodium (mmol/l) *U*_*k*_ urine potassium (mmol/l), *PEG* polyethylene glycol, *PEG-ELS* PEG/electrolyte lavage solution, *SP* sodium phosphate, *SPS/MC* sodium picosulfate/magnesium citrate, *Bis/Man* bisacodyl/mannitol

Renal water excretion is largely guided by AVP release. While its main determinant is plasma osmolality, several non-osmotic stimuli such as nausea and anxiety exist. In a case series involving 40 patients, raised AVP concentration after bowel cleansing but prior to endoscopy was found in 25% [20]. Therefore hyponatremia in this context has been traditionally regarded as a mere form of excessive AVP release [18, 20].

The importance of dietary solute intake is a lesser known facet of renal water excretion. A normal kidney can excrete urine with a concentration in the range of 50 mOsm/kg to 1200 mOsm/kg. Although the diluting capacity decreases with age, it generally allows for a broad range of water intake without the development of dilutional hyponatremia [22]. Up to 20 liters of water may be ingested over a 24-h period before serum sodium concentration begins to fall [23]. However, this assumes consumption of a normal diet containing adequate salt and protein. The average western diet yields approximately 800 mOsm of solutes per day, mainly derived from urea generation through protein metabolism, in addition to dietary electrolytes (mainly sodium and potassium) [24].

As demonstrated elegantly by Berl, urine excretion is dependent on solutes in urine that act as osmoles [22]. The maximum volume of urine excretion per day can be estimated by dividing the total daily solute excretion (roughly equal to the amount of solute intake) by the minimum urine osmolality in a given patient. If the minimum urine osmolality is 50 mosm/l (i.e. maximally dilute urine), then the daily urine volume will be 16 liters maximally (800 mOsm/day/50 mosm/kg = 16 l/day); in other words, the maximum water excretion per hour is approximately 700 ml. However, if sodium and overall food intake is limited, daily solute load can fall to 250 mOsm [23]. Under such circumstances, the maximum urine volume per day is only 5 liters (250/50), or about 200 ml per hour. If more fluid is ingested, particulary in a short period of time, dilutional hyponatremia will ensue. Consequently, restricted diets, when accompanied by copious water intake, can culminate in hyponatremia, with beer potomania constituting the prototypical example. Other forms of poor nutritional intake have been associated with hyponatremia, such as ovolactovegetarism, “tea and toast“diet“ of the elderly and “crash diets“ [25–27]. The underlying pathophysiological phenomenon has been dubbed “low solute hyponatremia“ [23]. Obviously, temporary food restriction is part of the preparation for colonoscopy; dietary regimens for colonic cleansing incorporate clear liquids and low-residue foods “during one to four days” [6].

Our patient’s diet on the two days preceeding her scheduled examination was virtually devoid of protein and sodium. Although we cannot specify exactly the amount of solutes ingested within the two days before admission in this case, it seems to be within the range of a previous case report [27].

The quantity and speed of water intake is another factor which merits emphasis. Although the manufacturer of PICOLAX ® recommends drinking “approximately 250 ml per hour of water or other clear fluid while the washout persists”, this advice was neither heeded by our patient nor was the recommended speed of fluid intake specified in the information leaflet [28]. Furthermore, leaflets occasionally instruct patients to “disregard the manufacturer’s instructions” [16]. Symptomatic hyponatremia can be induced by an acute water load in various scenarios, for instance prior to a sonographic examination or urine drug testing [29, 30]. As our patient ingested four liters of water within just two hours (about 70 ml/kg), her renal capacities for water elimination were obviously overwhelmed. Again, only one previous case report specifies the speed of fluid intake [9].

In theory, agents that induce an osmotic diarrhea should not allow relevant intestinal water absorption. However, there is anecdotal evidence of sodium and water retention after bowel preparation with PEG [31]. Furthermore, PICOLAX® is a combined formulation, one sachet containing magnesium oxide, sodium picosulfate and citric acid. While magnesium citrate is an osmotic laxative, sodium picosulfate is a pro-drug that is hydrolyzed by colonic bacteria to the active metabolite, which acts as a stimulant laxative [32]. Physiologically, most intestinal fluid is absorbed in the small bowel, consequently, rapid transit of the agent into the colon could allow subsequent water ingestion to be absorbed efficiently.

The patient presented was water intoxicated, but her urine was not maximally dilute, although urine osmolality was less than serum osmolality. Notably, low urine osmolality is not consistently found in low solute hyponatremia, as shown in a review involving 22 cases of beer potomania [24]. In our case, urine osmolality on admission is not known but a value of 232 mOsm/kg a few hours later is in keeping with ongoing AVP secretion, with various causes described above [20].

The brisk aquaresis after treatment with hypertonic saline renders support to our hypothesis that low solute intake contributed to the development of the hyponatremia. As described in cases of beer potomania or psychogenic polydipsia, the supplementation of osmotic agents (such as protein or salt) swiftly increases urine production, because the ratio of solute intake or excretion to urine osmolality determines urinary volume.

Lastly, NSAID are known to augment AVP effects and our patient’s diclofenac intake may have increased her vulnerability for water intoxication [5].

To avoid potentially devastating complications, we suggest to instruct patients at risk, in particular women above 50 years of age, to ensure liberal salt intake and sufficient protein intake on the days before endoscopy and to refrain from overdrinking, manifested in weight gain. Split-dose bowel-prep regimens, as opposed to day-before regimens, may be superior in this regard [33].

Hyponatremia has been dubbed “a forgotten consequence of bowel preparation for colonoscopy” [34]. When too much fluid is ingested in too little time, concomitant low dietary solute intake is a fundamental factor predisposing patients to “bowel prep hyponatremia”, particulary in the context of ongoing AVP secretion. Understanding that the cause of sodium imbalance is multifactorial is pivotal to recognizing and ideally preventing this complication.

## Abbreviations

AVP: Arginine vasopressin
BMI: Body mass index
CT: Computed tomography
NSAID: Nonsteroidal anti-inflammatory drug

## References

[1] Seeff LC, Richards TB, Shapiro JA (2004). How many endoscopies are performed for colorectal cancer screening? Results from CDC’s survey of endoscopic capacity. Gastroenterology.

[2] Connor A, Tolan D, Hughes S, Carr N, Tomson C (2012). Consensus guidelines for the safe prescription and administration of oral bowel-cleansing agents. Gut.

[3] Adrogué HJ, Madias NE (2000). Hyponatremia. N Engl J Med.

[4] Hsu YJ, Chiu JS, Lu KC, Chau T, Lin SH (2005). Biochemical and etiological characteristics of acute hyponatremia in the emergency department. J Emerg Med.

[5] Rosner MH, Kirven J (2007). Exercise-associated hyponatremia. Clin J Am Soc Nephrol.

[6] Wexner SD, Beck DE, Baron TH (2006). A consensus document on bowel preparation before colonoscopy: prepared by a task force from the American Society of Colon and Rectal Surgeons (ASCRS), the American Society for Gastrointestinal Endoscopy (ASGE), and the Society of American Gastrointestinal and Endoscopic Surgeons (SAGES). Dis Colon Rectum.

[7] Kim HG, Huh KC, Koo HS (2015). Sodium Picosulfate with Magnesium Citrate (SPMC) Plus Laxative Is a Good Alternative to Conventional Large Volume Polyethylene Glycol in Bowel Preparation: A Multicenter Randomized Single-Blinded Trial. Gut Liver.

[8] Lee SH, Cha JM, Lee JI (2015). Anaphylactic shock caused by ingestion of polyethylene glycol. Intest Res.

[9] Schröppel B, Segerer S, Keuneke C, Cohen CD, Schlöndorff D (2001). Hyponatremic encephalopathy after preparation for colonoscopy. Gastrointest Endosc.

[10] Ayus JC, Levine R, Arieff AI (2003). Fatal dysnatraemia caused by elective colonoscopy. BMJ.

[11] Frizelle FA, Colls BM (2005). Hyponatremia and seizures after bowel preparation: report of three cases. Dis Colon Rectum.

[12] Spengos K, Vassilopoulou S, Tsivgoulis G, Dimitrakopoulos A, Toulas P, Vassilapoulos D (2005). Hyponatraemia and central pontine myelinolysis after elective colonoscopy. Eur J Neurol.

[13] Nagler J, Poppers D, Turetz M (2006). Severe hyponatremia and seizure following a polyethylene glycol-based bowel preparation for colonoscopy. J Clin Gastroenterol.

[14] Dillon CE, Laher MS (2009). The rapid development of hyponatraemia and seizures in an elderly patient following sodium picosulfate/magnesium citrate (Picolax). Age Ageing.

[15] Baeg MK, Park JM, Ko SH, et al. Seizures due to hyponatremia following polyethylene glycol preparation; a report of two cases. Endoscopy. 2013; 45 Suppl 2 UCTN:E269-270. 10.1055/s-0033-1344568 24008460

[16] Veitenhansl M, Reisch N, Schmauss S, Wörnle M, Gärtner R (2007). Hyponatraemic encephalopathy and rhabdomyolysis. Complications after preparation for colonoscopy with mannitol. Internist (Berl).

[17] Más A, Chillarón JJ, Esteve E, Navalpotro I, Supervía A (2013). Severe rhabdomyolysis and hyponatremia induced by picosulfate and bisacodyl during the preparation of colonoscopy. Rev Esp Enferm Dig.

[18] Ko SH, Lim CH, Kim JY, Kang SH, Baeg MK, Oh HJ (2014). Case of inappropriate ADH syndrome: hyponatremia due to polyethylene glycol bowel preparation. World J Gastroenterol.

[19] Cho YS, Nam KM, Park JH, Byun SH, Ryu JS, Kim HJ (2014). Acute hyponatremia with seizure and mental change after oral sodium picosulfate/magnesium citrate bowel preparation. Ann Coloproctol.

[20] Cohen CD, Keuneke C, Schiemann U (2001). Hyponatraemia as a complication of colonoscopy. Lancet.

[21] Lichtenstein G (2009). Bowel preparations for colonoscopy: a review. Am J Health Syst Pharm.

[22] Berl T (2008). Impact of solute intake on urine flow and water excretion. J Am Soc Nephrol.

[23] Fenves AZ, Thomas S, Knochel JP (1996). Beer potomania: two cases and review of the literature. Clin Nephrol.

[24] Sanghvi SR, Kellerman PS, Nanovic L (2007). Beer potomania: an unusual cause of hyponatremia at high risk of complications from rapid correction. Am J Kidney Dis.

[25] Thaler SM, Teitelbaum I, Berl T (1998). “Beer potomania” in non-beer drinkers: effect of low dietary solute intake. Am J Kidney Dis.

[26] Yeates KE, Singer M, Morton AR (2004). Salt and water: a simple approach to hyponatremia. Review. CMAJ.

[27] Fox BD (2002). Crash diet potomania. Lancet.

[28] Ferring Pharmaceutical Limited (2016) PICOLAX ® package leaflet. Drayton Hall, United Kingdom.

[29] Tilley MA, Cotant CL (2011). Acute water intoxication during military urine drug screening. Mil Med.

[30] Klonoff DC, Jurow AH (1991). Acute water intoxication as a complication of urine drug testing in the workplace. JAMA.

[31] Granberry MC, White LM, Gardner SF (1995). Exacerbation of congestive heart failure after administration of polyethylene glycol-electrolyte lavage solution. Ann Pharmacother.

[32] Adamcewicz M, Bearelly D, Porat G, Friedenberg FK (2011). Mechanism of action and toxicities of purgatives used for colonoscopy preparation. Expert Opin Drug Metab Toxicol.

[33] Martel M, Barkun AN, Menard C, Restellini S, Kherad O, Vanasse A (2015). Split-Dose Preparations Are Superior to Day-Before Bowel Cleansing Regimens: A Meta-analysis. Gastroenterology.

[34] Scarpignato C, Blandizzi C (2014). Editorial: hyponatremia - a possible but forgotten consequence of bowel preparation for colonoscopy. Aliment Pharmacol Ther.

